# Tunable Dual-Wavelength with Twin-Pulse Dissipative Solitons in All-Normal Dispersion Yb-Doped Fiber Laser

**DOI:** 10.3390/mi13071049

**Published:** 2022-06-30

**Authors:** Xiaojun Zhu, Wen Liu, Yongquan Pan, Shuai Li, Guoan Zhang, Yancheng Ji, Li Zou, Zhipeng Liang, Juan Cao

**Affiliations:** 1 School of Information Science and Technology, Nantong University, Nantong 226019, China; zhuxj0122@ntu.edu.cn (X.Z.); 2110310010@stmail.ntu.edu.cn (W.L.); pyq7325@163.com (Y.P.); 2010310023@stmail.ntu.edu.cn (S.L.); gzhang@ntu.edn.cn (G.Z.); jiyancheng@ntu.edu.cn (Y.J.); zou.l@ntu.edu.cn (L.Z.); lzp1573@ntu.edu.cn (Z.L.); 2 Key Lab of Advanced Optical Manufacturing Technologies of Jiangsu Province & Key Lab of Modern Optical Technologies of Education Ministry of China, Soochow University, Suzhou 215006, China

**Keywords:** tunable wavelength, mode-locked, dissipative soliton, twin-pulse, Yb-doped fiber

## Abstract

A tunable dual-wavelength with two separated twin-pulse dissipative solitons (DSs) of Yb-doped mode-locked fiber laser in the all-normal-dispersion (ANDi) regime is firstly reported and demonstrated in this paper. A Sagnac loop is used as an all-fiber format spectral filter in the laser cavity, and stable twin-pulse DSs with different wavelength mode-locked lasers are achieved by the nonlinear polarization evolution (NPE) effect. By adjusting the polarization state of the Sagnac loop, the spectral ranges of the dual-wavelength can be tuned from 1031.3 nm to 1041.5 nm and from 1067.1 nm to 1080.9 nm, respectively. However, the pulse space between the two separated twin-pulse DSs is maintained, i.e., 41.63 ns. Furthermore, the twin-pulse can regress to the single-pulse when the pump power keeps dropping. It has been observed that the highest energy of the two twin-pulse DSs output is 23.36 nJ at a repetition rate of 2.282 MHz with a maximum pump power of 560 mW.

## 1. Introduction

Multi-wavelength and wavelength-tunable mode-locked fiber lasers have potential applications in the fields of optical fiber sensing, optics communications, and optical measurement instruments [1,2]. Various approaches have been exploited to achieve multi-wavelength by inserting several filters either into actively or passively mode-locked fiber laser cavity as wavelength selection devices, such as a birefringent filter, Mach–Zehnder interferometer, fiber Bragg grating, Sagnac loop filter, long-period grating, and fiber filter. In 2008, Tu et al., used a Mach–Zehnder interferometer to obtain stable eight lasing wavelengths in Yb-doped fiber laser [1]. In 2016, Hou et al., exploited a Sagnac loop to achieve wavelength-tunable passively mode-locked fiber laser [2]. For decades, multi-solitons have attracted significant attention due to their potential applications in micro-machining, high-resolution range-finding, geological exploration, and so on. The formation of multi-solitons is an essential topic in the domain of lasers. Various mechanisms could lead to forming the solitons, including the spectral filtering effect [3], the peak clamping effect [4], and the wave-breaking effect [5], as well as the soliton shaping of dispersive waves [6].

Recently, the ANDi systems have been developed significantly due to the higher pulse energy and simple cavity structure [7]. The dynamics of the DSs are handled by the normal cavity dispersion, cavity fiber nonlinearity, cavity gain, and cavity loss, i.e., the dissipative process. Depending on the ANDi cavity parameter selection, a variety of multiple DS nonlinear dynamics were observed, such as soliton rain [8], soliton molecules [9], breathing DS explosions [10], DS Resonance [11], bound-state solitons [12,13], twin-pulses [14], harmonic mode-locking [15] and double-scale pulses [16]. The formation of multiple DSs was caused by the peak-power-clamping effect [17], energy quantization [18], oversaturation of the nonlinear polarization evolution (NPE) [19,20], and so on. Among them, the spectral filter is a significant factor in the multiple DSs generations [3,21]. The selection of spectrum filters affected not only the characteristics of output pulse (such as pulse shaping, pulse quality, and pulse energy) but also the characteristics of output wavelengths (multi-wavelength, tunable wavelength) [7,22]. In 2010, Özgören et al., used a polarization-maintaining fiber (PMF) as the Lyot filter to obtain an all-fiber-format ANDi mode-locked femtosecond laser system [23]. In 2016, Denis et al., reported the cascaded generation of coherent Raman DSs with Lyot filters [24]. Luo et al., proposed multi-wavelength dissipative soliton (DS) generation in an all-normal-dispersion ytterbium-doped fiber laser based on a graphene-deposited tapered fiber [25].

So far, few theoretical and experimental investigations have been reported about the multi-pulse DSs generation in the ANDi laser with tunable output wavelength. In our previous work [15], twin-pulse DSs phenomena were observed in the Yb-doped fiber laser with a single wavelength. In 2019, Zhu et al., reported a tunable dual-wavelength mode-locking Er-doped fiber laser with two coherent solitons at different wavelengths [26]. Lin et al. observed tunable and switchable dual-wavelength DSs in a weak-birefringence ANDi Yb-doped fiber laser [27]. Zhang et al., proposed tunable single- and dual-wavelength DSs in an ANDi mode-locked Yb-doped fiber laser with an in-line birefringence fiber filter [28]. However, in all the reports above, each soliton has its corresponding wavelength output, i.e., one wavelength has only one pulse output. 

In this paper, a tunable dual-wavelength with two separated twin-pulse DSs for the different wavelengths is reported and demonstrated for the first time. The ANDi Yb-doped fiber laser is based on the NPE effect, with a Sagnac loop acting as the spectrum filter used for the dual-wavelength tuning. The Sagnac loop is fabricated by a 50:50 optical coupler (OC) connecting a 50 cm piece of single-mode fiber (SMF) with a 7.5 cm long PMF inserted and a polarization controller (PC) added. The SMF has an intensity-dependent loss, which contributes to an all-fiber format multi-wavelength filter in the cavity and leads to a steady multi-wavelength mode-locking output. By adjusting the polarization state of the Sagnac loop, the spectral range of the dual-wavelength can tune from 1031.1 nm to 1041.5 nm and from 1067.1 nm to 1080.9 nm, respectively. The maximum tunable range of the dual-wavelength is larger than ~13 nm. It is the first time to observe the two separated twin-pulse DSs in the ANDi system, as far as we have known. Furthermore, the number of pulses can be adjusted by changing the waveplates of the cavity, which has great potential applications in optical detections and optical sensing systems.

## 2. Experimental Setup

**Figure 1:**
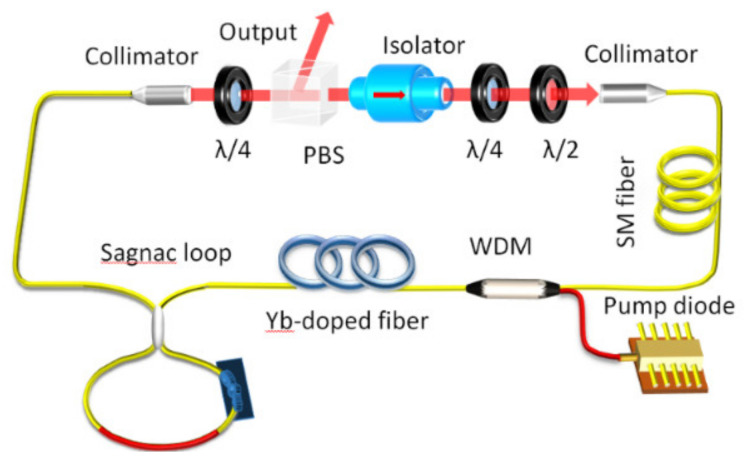
The configuration of an ANDi passively mode-locked Yb-doped fiber system with a Sagnac loop as the spectral filter. *λ*/4, *λ*/2: quarter and half-wave plates.

The experimental setup of the offered dual-wavelength with twin-pulse DSs Yb-doped fiber laser is shown in Figure 1. All fibers in the laser cavity are composed of pure ANDi fibers. A piece of Yb-doped fiber with a length of 28 cm is used for the gain-fiber (618 dB/m absorption at 976 nm) and is pumped by a laser diode with a maximum pump power of 560 mW and a central wavelength of 976 nm through a wavelength division multiplexer (WDM). The Sagnac loop plays the role of the spectrum filter and connects to one end of the WDM. A 72.4 m piece of SMF is employed in the cavity to increase the nonlinear effect. The nonlinear polarization evolution (NPE) effect is the passively mode-locked mechanism. It can be realized by a half-wave plate, a polarizing beam splitter (PBS), and two quarter-wave plates. The lasing is output from the NPE ejection port. The total cavity length of the laser system is ~78.3 m. The total group velocity dispersion (GVD) of the cavity is estimated +1.88 ps2 [7], and the total dispersion of the isolator, the waveplates, and the PBS were estimated to be +0.004 ps2. The wavelength of the output laser is monitored by an optical spectrum analyzer (Yokogawa AQ6370B), and the output pulses are transformed to electrical pulses by an 11 GHz photodetector, which can be measured by a 20 GHz digital storage oscilloscope (Tektronix DPO72004C) and detected by a radio-frequency (RF) spectrum analyzer (13.6 GHz bandwidth).

**Figure 2:**
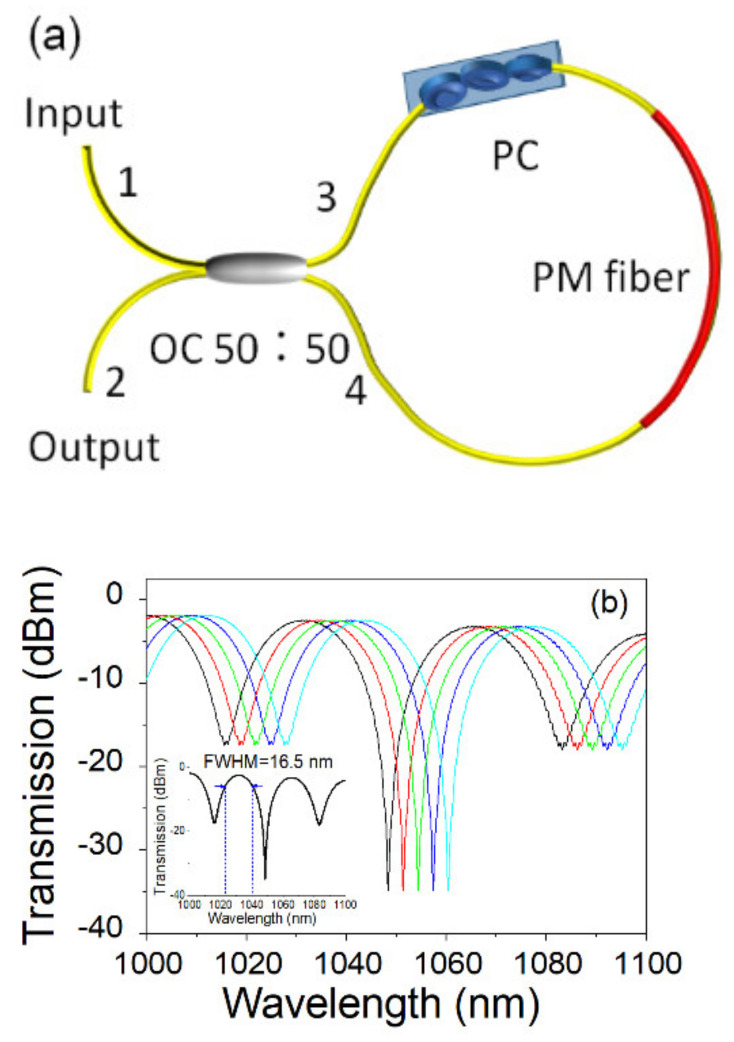
(**a**) Structure of the Sagnac loop with PM fiber; (**b**) Transmission spectra of Sagnac loop when the polarization controller change, inset: the initial state of the Sagnac loop.

The structural description of the Sagnac loop is illustrated in Figure 2a. A 7.5 cm long PMF connects two ends of a 50 cm piece of SMF, and a PC is inserted to change the polarization status of the cavity. When the light reaches 50:50 OC, it can be divided into two paths, i.e., a clockwise and an anti-clockwise path. Light passes the loop through two directions, which will form an interference phenomenon when they return to the original OC. Figure 2b shows the transmission spectra of the Sagnac loop when the polarization controller change. Two primary transmission channels are obtained in the wavelength range between 1000 nm to 1100 nm. The full-width at half maximum (FWHM) of each channel is 16.5 nm, and the isolation of each channel is higher than 17 dB, whereas the loss is less than 3.5 dB. From Figure 2, we can see that the Sagnac loop provided a good channel display, which can be used for a spectral filter in the ANDi system. Moreover, by adjusting the polarization controller in the Sagnac loop, the transmission spectra of the Sagnac loop will be tuned accordingly.

## 3. Results and Discussion

In the experiment, we use the Sagnac loop as the all-fiber format spectral filter for the dual-wavelength mode-locking output in the Yb-doped fiber laser. The threshold power of the laser is about 54 mW. When the pump power is stronger than the threshold, the lasing will be output from the NPE ejection port. By increasing the pump power, the dual-wavelength mode-locking laser can be obtained by altering the polarization state via the waveplates.

**Figure 3:**
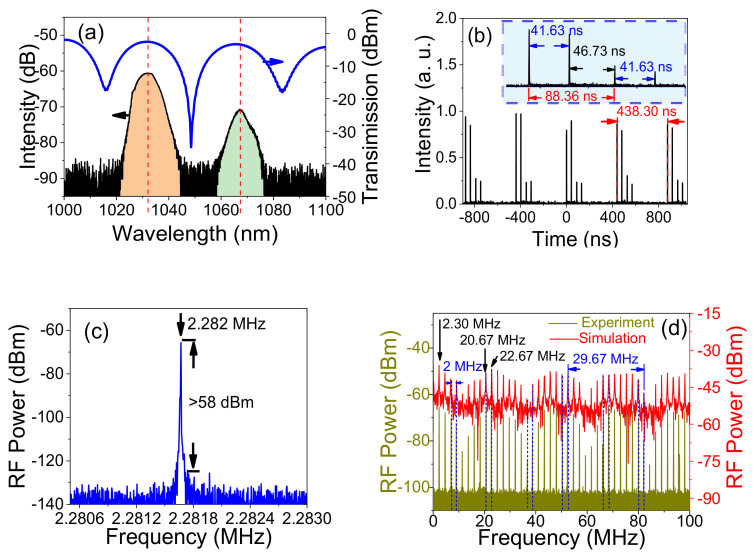
(**a**) Spectrum of the twin-pulse DSs and its comparison with the corresponding transmission spectrum of the Sagnac loop; (**b**) The mode-locked pulses train of the twin-pulse DSs; inset, the enlarge of the two twin-pluses DSs; (**c**) Radio-frequency spectrum of the two twin-pulse DSs with 2.5 KHz bandwidth; (**d**) Radio-frequency spectrum of the two twin-pulse DSs with 100 MHz bandwidth, measured by Radio-frequency analyzer (brown curve) and calculated from the pulse train measured by an oscilloscope (red curve).

Figure 3 shows the characteristics of the outputs of a dual-wavelength mode-locked laser with two twin-pulse DSs when the pump power is 560 mW. Figure 3a shows the comparison of the outputs between the dual-wavelength laser and the transmission spectrum of the Sagnac loop. From Figure 3a, we can see that the central wavelengths of the dual-wavelength ANDi fiber laser are 1031.3 nm and 1067.1 nm, which correspond to that of the Sagnac loop with 1031.8 nm and 1065.5 nm, respectively. The dual-wavelength lasers with the full width at half maximum (FWHM) are 8.1 nm and 5.5 nm, respectively. The shape of the dual-wavelength with DSs laser is approximately Gaussian-like, which is one of the typical characteristics of ANDi fiber lasers [22]. From Figure 3a, we can see that the spectrum is relatively smooth without fringe pattern or modulated trace, which shows the characteristics of the twin-pulse rather than the bound-state soliton by comparing with Ref. [24]. Figure 3b shows the pulse train of the dual-wavelength mode-locked laser measured by the high-speed photodiode and oscilloscope. It can be seen that two different intensities of twin-pulse DSs are obtained in the dual-wavelength mode-locked laser. Each twin-pulse DSs corresponds to an independent mode-locked wavelength, as shown in Figure 3a. Namely, the higher intensity twin-pulse corresponds to the wavelength with higher spectral energy, whereas the central wavelength is 1031.3 nm. The twin-pulse DSs with lower energy correspond to the spectrum with lower intensity, namely the central wavelength of 1067.1 nm. From Figure 3b, we can see that the pulse train period is ∼438.30 ns, which corresponds to the fundamental repetition frequency of 2.282 MHz, as shown in Figure 3c. In the inset figure of Figure 3b, the pulse–pulse spaces of the two twin-pulse DSs are both equal to 41.63 ns in the time domain. It is similar to the phenomenon that soliton pairs were oscillating at fixed positions, as discussed in Refs. [12,13]. The time distance between the two twin-pulse DSs is about ~88.36 ns, which corresponds to the fact that the spectrum interval of dual-wavelength is ~35.8 nm. Interestingly, we can calculate the energy for each twin-pulse DSs by the integration methods as shown in Figure 3a [26]. The average power of the two twin-pulse DSs accounts for ∼73.1% and ~26.9%, respectively. When the maximum pump power is 560 mW, the average power of the outputs measured is 53.3 mW, and the highest energy of output pulses is 23.36 nJ. Thus, the average power of the separated twin-pulse DSs with the central wavelength of 1031.3 nm and 1067.1 nm are 42.27 mW and 11.03 mW, respectively. Therefore, the average energy of the two separated twin-pulse DSs can be deduced by 18.52 nJ and 4.84 nJ, respectively.

In order to confirm the stability of the solitons, the RF spectra are measured, as shown in Figure 3c. The fundamental repetition frequency (FRF) of the laser output is 2.282 MHz, which corresponds to the lengths of the laser cavity of ~78.3 m, and the signal-to-noise ratio (SNR) at the FRF is larger than ∼58 dB, with a 5 Hz resolution bandwidth (RBW) and 2.5 kHz span. Figure 3d shows the RF spectrum with 100 MHz bandwidth and an RBW of 167 kHz. It should be noted that two different frequency intervals have coexisted in the RF spectrum. They are interleaved distribution and simultaneously oscillated in the RF spectrum. The first one is 2.3 MHz, which is the normal distribution, and the second one is 2.0 MHz, which is the periodic distribution in the RF spectrum (with the blue dash in Figure 3d). Because of the two different frequency elements, two modulation envelopes coexist in the RF spectrum with the periods of 22.67 MHz and 20.67 MHz, and they correspond to the time spacing of 41.63 ns and 46.73 ns in the pulses train, as shown in Figure 3b, respectively. To confirm the correction of the measurements, we compared the RF spectrum of the pulse train data by using a Fourier transform algorithm with the RF spectrum measured by the RF spectrum analyzer, as shown in Figure 3d. We can see they have a good agreement with each other. From Figure 3, we can see that the twin-pulses DSs have a narrow spectral bandwidth and a fixed repetition rate. All the above phenomena can confirm that the twin-pulse output observed in our experiment is mode-locked rather than noise-like pulses [21].

**Figure 4:**
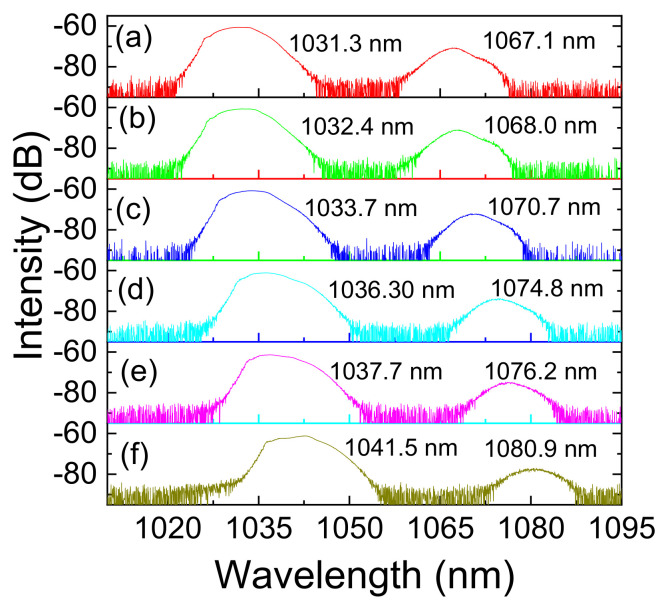
Tunable spectra of the dual-wavelength mode-locking operation by changing the polarization state of the Sagnac loop. (**a**) The central wavelengths of the dual-wavelength mode-locked laser are 1031.3 nm and 1067.1 nm, respectively; (**b**) The central wavelengths are 1032.4 nm and 1068.0 nm, respectively; (**c**) The central wavelengths are 1033.7 nm and 1070.7 nm, respectively; (**d**) The central wavelengths are 1036.30 nm and 1074.8 nm, respectively; (**e**) The central wavelengths are 1037.7 nm and 1076.2 nm, respectively; (**f**) The central wavelengths are 1041.5 nm and 1080.9 nm, respectively.

Figure 4 shows the dual-wavelength mode-locking output at various wavelengths in the ANDi regime. During the tuning process, the pump power keeps at 474 mW, and the mode-locking mode maintains without readjusting any elements in the system. When adjusting the PC of the Sagnac loop, the spectra of the dual-wavelength outputs are shifted to longer wavelengths, i.e., from 1031.3 nm to 1041.5 nm for output wavelength one and from 1067.7 nm to 1080.9 nm for wavelength two, respectively. The maximum ranges of the dual-wavelength tuning are 10 nm and 13 nm, respectively. This is the first time to achieve the continuously tunable mode-locked dual-wavelength output with twin-pulse DSs in different wavelengths in the ANDi system. During the entire tuning process, the output power gradually decreases from 41.2 mW to 33.4 mW when the pump power keeps at 474 mW. The spectra intensity of the one in the longer wavelength of the dual-wavelength decreases from ~−70 dB to ~−77 dB, as shown in Figure 4a and in Figure 4f, respectively, which is due to the limitation of the bandwidth of gain medium and the bandwidth of the Sagnac loop filter during the tuning process. It should be noted that when the polarization state of the Sagnac loop is changed, the wavelengths of the mode-locked output are tuned accordingly without adjusting any other polarization components in the laser cavity, such as the quarter and half-wave plates. This is totally different from the mechanism reported previously [29], in which the wavelength tunability was based on the birefringent states in the cavity. For different required wavelengths, the birefringent and wave plates must be readjusted for mode-locking, which is very complex and impractical in industrial applications. Here, we only need to change the polarization state of the Sagnac loop to tune the mode-locked wavelength continuously in a well-controlled and repeatable manner.

**Figure 5:**
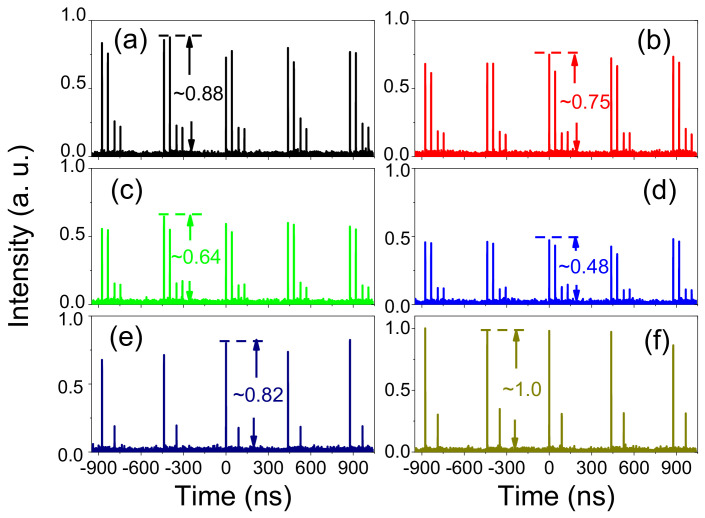
Output pulse evolving process when the cavity parameters changes; (**a**) the twin-pulse DSs with a pump power of 560 mW; (**b**) the twin-pulse DSs with a pump power of 520 mW; (**c**) the twin-pulse DSs with a pump power of 474 mW; (**d**) the twin-pulse DSs with a pump power of 430 mW; (**e**) the single-pulse DS with a pump power of 430 mW; (**f**) the single-pulse DS with polarization state changing.

Different types of laser pulses outputs in the ANDi long cavity Yb-doped fiber laser are generated by changing the pump power alone, as shown in Figure 5. An example is shown in Figure 5b; two twin-pulse DSs are generated when the pump power is increased to 520 mW. This phenomenon can be explained by the spectral filter and oversaturation of the NPE characteristics in the system [15,19,20]. It is known that NPE, in contrast to the saturable absorber (SA), has a sinusoidal dependence of transmittance on intensity. The transmittance of SA for a given system is limited to a value that can be changed by modulation depth and nonlinear cavity loss via adjusting waveplates [21]. When the accumulated pulse power is larger than the value of maximal SA intensity (called oversaturation of the NPE), the pulse will split up, i.e., multi-pulse soliton or high harmonic oscillation occurs. The Sagnac loop as the spectral filter is another reason for the formation of multi-pulse or harmonically mode-locked (HML) pulses. This has been demonstrated and reported in [3] and [21], in which the multiple pulses appeared or disappeared when the pump power was increased or decreased. Figure 5 also shows the evolving process of the two twin-pulse DSs output when the pump power changes. It is demonstrated in our experiment that the two-pulse DSs in the system can be recognized as a “single DS” when the two-pulse DSs are tightly like a bunching mode at a fixed position and move together as a whole in the laser cavity. As shown in Figure 5, when the pump power is 560 mW, a stable mode-locking state is working with two twin-pulse DSs output, and the output power is 47.4 mW. When the pump power decreased from the 560 mW to 430 mW, the average intensity of the two twin-pulse DSs with higher energy twin-pulse decreased from ~0.88 to ~0.48, and the output power decreased from 47.4 mW to 23.4 mW, as shown in Figure 5a–d, respectively. By continuously reducing the pump power, the evolving process of the twin-pulse DSs is observed. When the pump power is lower than a certain value, the twin-pulse DSs phenomenon disappears. As shown in Figure 5e, the twin-pulse DSs change to a single-pulse DS in each wavelength when the pump power is lower than 430 mW. It is found that when the pump power is reduced from 560 mW to 430 mW, the mode-locking state of the twin-pulse DSs is still stable, whereas the intensity starts decreasing. When the pump power reduces to about 430 mW, the twin-pulse mode-locked states become unstable. When the pump power is lower than 430 mW, the twin-pulse DSs change to single-pulse DSs. However, the maximum output intensity of the DS increases from ~0.48 to ~0.82, and the output power also increases from 23.4 mW to 45.0 mW as shown in Figure 5d,e, respectively.

It is shown that the energy of the twin-pulse DSs drops with the decrease of the pump power. When the pump power reduces to 430 mW, the energy in the cavity is not enough to maintain the twin-pulse mode-locking state, so the twin-pulse begins to jitter sharply. As long as the power is reduced slowly, the number of pulses will change. When the number of the DS decreased to single, the mode-locking state returned to stable again. While the pump power drops continually, the output power reduces correspondingly. When the pump power is lower than 248 mW, the mode-locked state will disappear. However, when gradually increasing the pump power, it gradually begins to achieve the mode-locking state again; the pump power needs to reach 300 mW. It is the hysteresis phenomenon in the multi-pulse formation [30]. From Figure 5e,f, we can see that by adjusting the wave plates to change the SA in the cavity, the intensity of the single-pulse DS in each wavelength is increased from ~0.82 to ~0.1, and the outputs power also increase from 45.0 mW to 48.5 mW. Continually changing the SA, the twin-pulse DSs will be obtained again by adjusting the waveplates. It is caused by the phenomenon of energy quantization in the laser system. Therefore, From Figure 5, we can see that the twin-pulse DSs have the same properties of energy quantization and pump power hysteresis in comparison with the single-pulse solitons [18].

**Figure 6:**
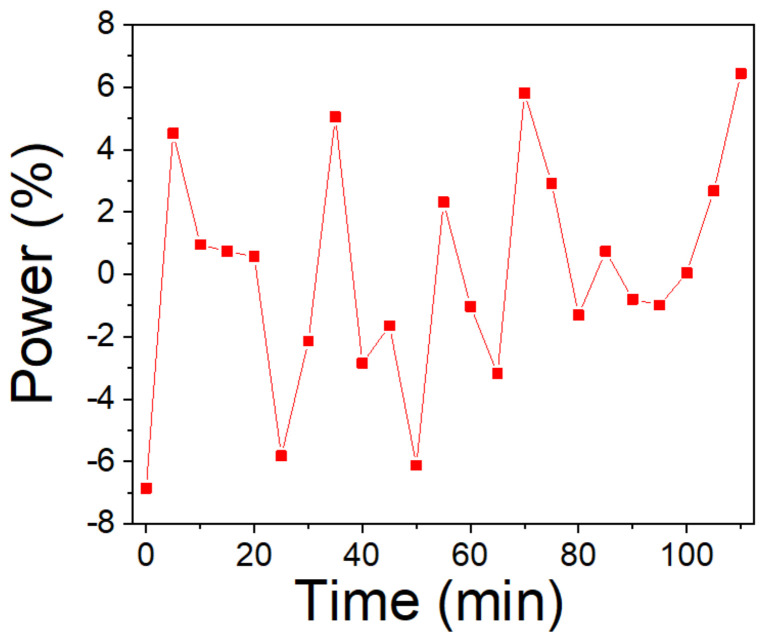
Measurement of output power fluctuation.

To confirm the stability of the output laser, we use a power meter to measure the output power of the dual-wavelength with a twin-pulse mode-locked laser for 110 min, and the results are shown in Figure 6. When the pump power was 520 mW, the average output power was ~39 mW. We can see that there is a slight jitter during the measurement period, and the maximum peak power fluctuation is within the degree of ~ 7%. It is found that the system can work for days without degradation in power, output spectrum, or state of the mode-locking under the condition of constant temperature and humidity.

## 4. Conclusions

In summary, a tunable dual-wavelength mode-locked laser with two different twin-pulse DSs for each wavelength has been demonstrated. An all-fiber format Sagnac-loop acting as the spectral filter is explored in the ANDi cavity. The central wavelength spectra of the output lasers were solely determined by the transmission of the spectral passband of the Sagnac loop. By changing its polarization state via adjusting the waveplates in the cavity, the dual-wavelength mode-locking state achieves tuned spectra range from 1031.1 nm to 1041.5 nm and from 1067.1 nm to 1080.9 nm, respectively. In the wavelength tuning process, the two different twin-pulse DSs outputs were steadily maintained. Furthermore, the highest energy of the two twin-pulse DSs output was 23.36 nJ at a repetition rate of 2.282 MHz with a maximum pump power of 560 mW. By adjusting the polarization controller in the Sagnac loop, the dual-wavelength outputs can be tuned with the mode-locked station keeping. The approach of steady twin-pulse DSs offers new possibilities for nonlinear fundamental research as well as for applications in multi-comb frequency, multi-pulses in nonlinear detections, and so on.

## Data Availability

Data available on request due to restrictions eg privacy or ethical. The data presented in this study are available on request from the corresponding author.

## References

[1] Tu C., Guo W., Li Y., Zhang S., Zhu H., Lu F. (2008). Multiwavelength Yb-doped fiber ring laser based on a Mach-Zehnder interferometer. Microw. Opt. Technol. Lett..

[2] Hou L., Li M., He X., Lin Q., Guo H., Lu B., Qi X., Chen H., Bai J. (2016). Wavelength-tunable dissipative pulses from Yb-doped fiber laser with Sagnac filter. Laser Phys. Lett..

[3] Komarov A., Komarov K., Leblond H., Sanchez F. Spectral management of solitons interaction and generation regimes of fiber laser. Proceedings of the 2007 9th International Conference on Transparent Optical Networks.

[4] Zhao L., Tang D., Cheng T., Tam H., Lu C. (2007). Generation of multiple gain-guided solitons in a fiber laser. Opt. Lett..

[5] Martel G., Chedot C., Reglier V., Hideur A., Ortaç B., Grelu P. (2007). On the possibility of observing bound soliton pairs in a wave-breaking-free mode-locked fiber laser. Opt. Lett..

[6] Tang D., Zhao B., Zhao L., Tam H. (2005). Soliton interaction in a fiber ring laser. Phys. Rev. E.

[7] Chong A., Renninger W., Wise F. (2007). All-normal-dispersion femtosecond fiber laser with pulse energy above 20 nJ. Opt. Lett..

[8] Bao C., Xiao X., Yang C. (2013). Soliton rains in a normal dispersion fiber laser with dual-filter. Opt. Lett..

[9] Peng J., Zhan L., Luo S., Shen Q. (2013). Generation of soliton molecules in a normal-dispersion fiber laser. IEEE Photonics Technol. Lett..

[10] Peng J., Zeng H. (2019). Experimental observations of breathing dissipative soliton explosions. Phys. Rev. Appl..

[11] Chowdhury S., Pal A., Chatterjee S., Sen R., Pal M. (2018). Multipulse Dynamics of Dissipative Soliton Resonance in an All-Normal Dispersion Mode-Locked Fiber Laser. J. Lightwave Technol..

[12] Zhao B., Tang D., Shum P., Guo X., Lu C., Tam H. (2004). Bound Twin-Pulse Solitons in a Fiber Ring Laser. Phys. Rev. E.

[13] Yun L., Liu X. (2012). Generation and Propagation of Bound-State Pulses in a Passively Mode-Locked Figure-Eight Laser. IEEE Photonics J..

[14] Zhu X., Wang C., Liu S., Zhang G. (2012). Tunable high-order harmonic mode-locking in Yb-doped fiber laser with all-normal dispersion. IEEE Photonics Technol. Lett..

[15] Zhu X., Bu C., Wang C., Xu R., Zhang G. (2015). Harmonically mode-locked twin-pulse dissipative solitons Yb-doped fiber laser. IEEE Photonics Technol. Lett..

[16] Kobtsev S., Kukarin S., Smirnov S., Turitsyn S., Latkin A. (2009). Generation of double-scale femto/pico-second optical lumps in mode-locked fiber lasers. Opt. Express.

[17] Lin W., Wang S., Zhao Q., Chen W., Gan J., Xu S., Yang Z. (2015). Mechanism of solitary wave breaking phenomenon in dissipative soliton fiber lasers. Opt. Express.

[18] Renninger W., Chong A., Wise F. (2010). Area theorem and energy quantization for dissipative optical solitons. J. Opt. Soc. Am. B.

[19] Zhang L., Zhuo Z., Pan Z., Wang Y., Zhao J., Wang J. (2013). Investigation of pulse splitting behaviour in a dissipative soliton fibre laser. Laser Phys. Lett..

[20] Martel G., Chédot C., Hideur A., Grelu P. (2008). Numerical maps for fiber lasers mode locked with nonlinear polarization evolution: Comparison with semi-analytical models. Fiber Integr. Opt..

[21] Zhao L., Tang D. (2006). Generation of 15-nJ bunched noise-like pulses with 93-nm band width in an erbium-doped fiber ring laser. Appl. Phys. B.

[22] Bale B., Kutz J., Chong A., Renninger W., Wise F. (2008). Spectral filtering for mode locking in the normal dispersive regime. Opt. Lett..

[23] Özgören K., Ilday F. (2010). All-fiber all-normal dispersion laser with a fiber-based Lyot filter. Opt. Lett..

[24] Kharenko D., Bednyakova A., Podivilov E., Fedoruk M., Apolonski A., Babin S. (2016). Cascaded generation of coherent Raman dissipative solitons. Opt. Lett..

[25] Luo Z., Huang Y., Wang J., Cheng H., Cai Z., Ye C. (2012). Multiwavelength dissipative-soliton generation in Yb-fiber laser using graphene-deposited fiber-taper. IEEE Photonics Technol. Lett..

[26] Zhu T., Wang Z., Wang D., Yang F., Li L. (2019). Generation of wavelength-tunable and coherent dual-wavelength solitons in the C + L band by controlling the intracavity loss. Photonics Res..

[27] Lin H., Guo C., Ruan S., Yang J., Ouyang D., Wu Y., Wen L. (2013). Tunable and switchable dual-wavelength dissipative soliton operation of a weak-birefringence all-normal-dispersion Yb-doped fiber laser. IEEE Photonics J..

[28] Zhang Z., Xu Z., Zhang L. (2012). Tunable and switchable dual-wavelength dissipative soliton generation in an all-normal-dispersion Yb-doped fiber laser with birefringence fiber filter. Opt. Express.

[29] Schultz M., Karow H., Wandt D., Morgner U., Kracht D. (2009). Ytterbium femtosecond fiber laser without dispersion compensation tunable from 1015 nm to 1050 nm. Opt. Commun..

[30] Komarov A., Leblond H., Sanchez F. (2005). Multistability and Hysteresis Phenomena in Passively Mode-Locked Fiber Lasers. Phys. Rev. A.

